# Supplementary material for: Experimental and Computational Study on Motor Control and Recovery After Stroke: Toward a Constructive Loop Between Experimental and Virtual Embodied Neuroscience
Source: Front Syst Neurosci. 2020 Jul 7;14:31. doi: 10.3389/fnsys.2020.00031 (PMC7359878; doi:10.3389/fnsys.2020.00031)
Supplement: Supplementary file 1 [file Data_Sheet_1.PDF]

Supplementary Material

1 SUPPLEMENTARY TABLES AND FIGURES

1.1 Tables

	parameter	value
muscle spindle	N	60
	a_{dyn}	0.08
	a_{st}	0.06
	$bag1_G$	$20\,000.0$
	$bag1_{Kpr}$	0.15
	$bag1_{Ksr}$	10.4649
	$bag1_{Lpr0}$	0.76
	$bag1_{LprN}$	0.0
	$bag1_{Lsec}$	0.0
	$bag1_{Lsr0}$	0.04
	$bag1_{LsrN}$	0.0423
	$bag1_R$	0.46
	$bag1_X$	0.0
	$bag1_a$	$3.333\,333$
	$bag1_{beta}$	0.2592
	$bag1_{beta0}$	0.0605
	$bag1_{gamma}$	0.0289
	$bag2_G$	$10\,000.0$
	$bag2_{Kpr}$	0.15
	$bag2_{Ksr}$	10.4649
	$bag2_{Lpr0}$	0.76
	$bag2_{LprN}$	0.89
	$bag2_{Lsec}$	0.04
	$bag2_{Lsr0}$	0.04
	$bag2_{LsrN}$	0.0423
	$bag2_R$	0.46
	$bag2_X$	0.7
	$bag2_a$	$3.333\,333$
	$bag2_{beta}$	-0.046
	$bag2_{beta0}$	0.0822
	$bag2_{gamma}$	0.0636
	$beta_{Ca}$	0.001
	$chain_G$	$10\,000.0$
	$chain_{Kpr}$	0.15
	$chain_{Ksr}$	10.4649
	$chain_{Lpr0}$	0.76
	$chain_{LprN}$	0.89
	$chain_{Lsec}$	0.04

	$chain_{Lsr0}$	0.04
	$chain_{LsrN}$	0.0423
	$chain_R$	0.46
	$chain_X$	0.7
	$chain_a$	$3.333\,333$
	$chain_{beta}$	-0.069
	$chain_{beta0}$	0.0822
	$chain_{gamma}$	0.0954
	tau_{dyn}	310.0
	tau_{st}	425.0
interneurons	N	196
	C_m	250.0
	Ca	0.0
	E_L	-70.0
	I_e	0.0
	V_{reset}	-70.0
	V_{th}	-55.0
	$beta_{Ca}$	0.001
	t_{ref}	2.0
	tau_{Ca}	$10\,000.0$
	tau_m	10.0
	tau_{syn-ex}	2.0
	tau_{syn-in}	2.0
muscle model/α-motoneurons	N	196
	d_{max}	8.5
	d_{min}	120.0
	D_{SF}	9.11×10^{-7}
	c_{spf}	1×10^{-2}
	τ_{max}	12.5×10^{-3}
	τ_{adj}	60×10^{-6}
	τ_{slp}	94.4
	p_{max}	15.0
	p_{min}	2.25
	F_{SF}	1.21
	s_{min}	0.104
	s_{sl}	0.207
	T_{SF}	0.6892
	Ca	0.0
	E_L	-70.0
	I_e	0.0
	V_{reset}	-70.0
	V_{th}	-55.0
	$beta_{Ca}$	0.001
	t_{ref}	2.0
	tau_{Ca}	$10\,000.0$

	tau_{syn-ex}	2.0
	tau_{syn-in}	2.0
synaptic connections	weight	0.85
	delay	0.1
	probability	0.6
descending connections	weight	1.8
	delay	1.0
	probability	0.9

Table S1: Parameters of the spinal cord circuitry.

muscle	parameter	value
$Radius_1$	f_o^M	0.1
	ℓ_o^M	0.006 026
	ℓ^M	0.006 026
	ℓ^T	0.000 603
	α_o	0.0
$Radius_2$	f_o^M	0.1
	ℓ_o^M	0.005 879
	ℓ^M	0.005 879
	ℓ^T	0.000 588
	α_o	0.0
$Humerus_1$	f_o^M	0.14
	ℓ_o^M	0.0122
	ℓ^M	0.0122
	ℓ^T	0.001 22
	α_o	0.0
$Humerus_2$	f_o^M	0.14
	ℓ_o^M	0.010 242
	ℓ^M	0.010 242
	ℓ^T	0.001 024
	α_o	0.0

Table S2: Parameters of the musculoskeletal embodiment.
